# Homers at the Interface between Reward and Pain

**DOI:** 10.3389/fpsyt.2013.00039

**Published:** 2013-06-07

**Authors:** Ilona Obara, Scott P. Goulding, Adam T. Gould, Kevin D. Lominac, Jia-Hua Hu, Ping Wu Zhang, Georg von Jonquieres, Marlin Dehoff, Bo Xiao, Peter H. Seeburg, Paul F. Worley, Matthias Klugmann, Karen K. Szumlinski

**Affiliations:** ^1^Department of Psychology, Neuroscience Research Institute, University of California at Santa BarbaraSanta Barbara, CA, USA; ^2^School of Medicine, Pharmacy and Health, Queen’s Campus, University of DurhamStockton on Tees, UK; ^3^Department of Neuroscience, Johns Hopkins University School of MedicineBaltimore, MD, USA; ^4^Translational Neuroscience Facility, School of Medical Sciences, UNSW Kensington Campus, University of New South WalesSydney, NSW, Australia; ^5^Department of Molecular Neurobiology, Max Planck Institute Medical ResearchHeidelberg, Germany

**Keywords:** Homer proteins, Group1 metabotropic glutamate receptors, NMDA receptors, neuropathic pain, heroin, nucleus accumbens, conditioned place-preference, conditioned place-aversion

## Abstract

Pain alters opioid reinforcement, presumably *via* neuroadaptations within ascending pain pathways interacting with the limbic system. Nerve injury increases expression of glutamate receptors and their associated Homer scaffolding proteins throughout the pain processing pathway. Homer proteins, and their associated glutamate receptors, regulate behavioral sensitivity to various addictive drugs. Thus, we investigated a potential role for Homers in the interactions between pain and drug reward in mice. Chronic constriction injury (CCI) of the sciatic nerve elevated Homer1b/c and/or Homer2a/b expression within all mesolimbic structures examined and for the most part, the Homer increases coincided with elevated mGluR5, GluN2A/B, and the activational state of various down-stream kinases. Behaviorally, CCI mice showed pain hypersensitivity and a conditioned place-aversion (CPA) at a low heroin dose that supported conditioned place-preference (CPP) in naïve controls. Null mutations of *Homer1a*, *Homer1*, and *Homer2*, as well as transgenic disruption of mGluR5-Homer interactions, either attenuated or completely blocked low-dose heroin CPP, and none of the CCI mutant strains exhibited heroin-induced CPA. However, heroin CPP did not depend upon full Homer1c expression within the nucleus accumbens (NAC), as CPP occurred in controls infused locally with small hairpin RNA-Homer1c, although intra-NAC and/or intrathecal cDNA-Homer1c, -Homer1a, and -Homer2b infusions (to best mimic CCI’s effects) were sufficient to blunt heroin CPP in uninjured mice. However, arguing against a simple role for CCI-induced increases in either spinal or NAC Homer expression for heroin CPA, cDNA infusion of our various cDNA constructs either did not affect (intrathecal) or attenuated (NAC) heroin CPA. Together, these data implicate increases in glutamate receptor/Homer/kinase activity within limbic structures, perhaps outside the NAC, as possibly critical for switching the incentive motivational properties of heroin following nerve injury, which has relevance for opioid psychopharmacology in individuals suffering from neuropathic pain.

## Introduction

Comorbidity exists between chronic pain and motivational disturbances (e.g., Doth et al., 2010; Ohayon and Schatzberg, 2010; Jarcho et al., 2012; Oluigbo et al., 2012), and a cause-effect relationship between chronic pain and a blunted motivational state is apparent also in animal studies (c.f., Niikura et al., 2010). The pain processing pathway interacts at multiple levels with brain structures embedded within mesocorticolimbic subcircuits underpinning subjective responses to, as well as the incentive value of, stimuli (both appetitive or noxious), including subregions of the prefrontal cortex (PFC), nuclei of the amygdala (AMY), the ventral tegmental area (VTA), and subregions of the nucleus accumbens (NAC) (c.f., Leknes and Tracey, 2008; Becker et al., 2012). While the neurocircuitry underpinning pain perception and the subjective pain response is known to involve activation within several frontal cortical subregions and thalamus (c.f., Leknes and Tracey, 2008; Oluigbo et al., 2012), the precise neurocircuitry involved in pain-induced alterations in motivation are less well understood (Becker et al., 2012).

Patients’ hypersensitivity to pain stimuli correlates with increases in PFC-NAC connectivity in recent neuroimaging studies and, importantly, heighted connectivity is predictive of affective pain, as well as pain severity in humans (e.g., Baliki et al., 2010, 2012). In animal and human studies, noxious stimuli, including chronic constriction injury (CCI) of the sciatic nerve, alters the activational state of mesocorticolimbic circuit (e.g., Kuroda et al., 1995; Rodella et al., 1998; Narita et al., 2003, 2005; Ozaki et al., 2003, 2004; Wood et al., 2007). Thus, injury-induced mesocorticolimbic anomalies are theorized to underpin the negative affective aspects of pain, as well as the impairments in motivation often observed in individuals suffering from chronic somatic pain (c.f., Leknes and Tracey, 2008; Becker et al., 2012; Oluigbo et al., 2012). In support of an interaction between a chronic pain state and drug reinforcement/reward, there is an absence of both opioid drug- and psychomotor stimulant-induced conditioned place-preference (CPP) in animal models of inflammatory or neuropathic pain (c.f., Niikura et al., 2010), which is consistent with very little evidence for the clinical diagnosis of addiction in individuals undergoing pharmacotherapy for chronic pain symptoms (e.g., Niikura et al., 2010; Minozzi et al., 2013). However, pain symptoms augment opioid drug consumption under operant procedures in animal models, which is theorized to reflect a compensation for a depressed mesocorticolimbic circuit (Colpaert et al., 1982, 2001; Dib and Duclaux, 1982; Lyness et al., 1989; Martin et al., 2007, 2011), fitting with extant CPP data indicating blunted drug-conditioned reward following nerve injury (c.f., Niikura et al., 2010).

Glutamate neuroadaptations within the mesocorticolimbic system are theorized to contribute significantly to drug reward/reinforcement in various addiction-related animal models (e.g., Szumlinski et al., 2008; Kalivas, 2009; Olive et al., 2012). As noxious, painful stimuli augment glutamatergic neurotransmission both at the spinal and supraspinal levels and glutamatergic hyperactivity is considered an active mediator of pain symptomatology (c.f., Chiechio and Nicoletti, 2012; Harris and Clauw, 2012; Wozniak et al., 2012; Osikowicz et al., 2013), the present study employed a combination of immunoblotting and behavioral genetic approaches to test the hypothesis that injury-induced increases in mesocorticolimbic glutamate transmission contribute to a blunted motivational state within the confines of a heroin CPP model of drug reward.

## Materials and Methods

### Subjects

Subjects included adult male C57BL/6J (B6) mice (8 weeks of age; 25–30 g; the Jackson Laboratories, Bar Harbor, ME, USA), as well as several strains of constitutive gene knock-out (KO) mice that were available at the time of study, including *Homer1a* KO (Hu et al., 2012), *Homer1* KO (Yuan et al., 2003), and *Homer2* KO (Shin et al., 2003) mice. Knock-in (KI) mice expressing mutant mGluR5 with a phenylalanine (F) to arginine (R) switch at position 1128 that markedly reduces mGluR5-Homer interactions (*Grm5^R/R^*; Cozzoli et al., 2009) were also employed. All the above mutant strains were bred in-house at UCSB from mating of heterozygous breeder pairs (B6 × 129Xi/SvJ background) and male wild-type (WT), heterozygous (HET), and homozygous KO/KI littermate pups were employed in all studies. For the KO/KI strains bred in-house, mice were selected from a minimum of four different litters within each replicate and testing began at 7–8 weeks of age. Experimental protocols were approved by the IACUCs of our respective institutions and were consistent with the guidelines provided by NIH and the Committee for Research and Ethical Issues of IASP.

### Neuropathic pain, inflammatory pain, and pain threshold assessment

The procedures for inducing peripheral neuropathy by CCI of the sciatic nerve were identical to those described recently by our group (Obara et al., 2013). The total length of nerve affected was 3–4 mm. Mechanical and cold hypersensitivity at the plantar surface of the hind paw ipsilateral to the injury was assessed, respectively, using von Frey filaments (0.07–6 g; Stoelting, Wood Dale, IL, USA) and the acetone test (50 μl) before nerve injury (as one index of basal pain threshold), and on days 3, 7, and/or 14 post-CCI (e.g., Obara et al., 2003, 2013; Osikowicz et al., 2008).

### Immunoblotting

At 1 or 2 weeks after nerve injury, the entire NAC, the VTA, the entire AMY, and the PFC (anterior cingulate, prelimbic, and infralimbic cortices) were dissected from B6 mice (*n* = 6–8/group/time-point) over ice, homogenized in a buffer containing both protease and phosphatase inhibitors and subjected to conventional immunoblotting procedures (20 μg protein/lane) as described previously by our group (e.g., Goulding et al., 2011; Obara et al., 2013). The details regarding the antibodies employed to detect protein levels of Homer1b/c, Homer2a/b, mGluR1, mGluR5, GluN2A, GluN2B, PI3K, p(Tyr)PI3K p85α binding motif, ERK1/2, p(Tyr204)ERK1/2, PKCε, p(Ser729)PKCε, and calnexin (loading and transfer control) are provided in the legend for Figure 2. The data for neuropathic animals at the different time-points post-injury were expressed as a percent change from the mean signal of the uninjured controls for each individual membrane (*n* = 3–4/membrane) as published previously (e.g., Obara et al., 2013).

### Heroin-induced place-conditioning

Mice were assayed for the development of heroin place-conditioning, starting at 14 days post-nerve injury. The apparatus and procedures for heroin place-conditioning were similar to those employed in our previous studies of drug-conditioned reward in mice (e.g., Penzner et al., 2008) and proceeded in the following four sequential phases: habituation, preconditioning test (Pre-Test), conditioning, postconditioning test (Post-test). All sessions were 15 min in duration and animals received no injections during the habituation, Pre-Test, or Post-Test sessions when they had free-access to both compartments of the apparatus. For conditioning, mice received four alternating pairings of distinct compartments with either intraperitoneal heroin (0.01–3 mg/kg; vol = 0.01 ml/kg) or an equivalent volume of saline in an unbiased fashion. Locomotor activity was monitored during all free-access sessions, as well as on the first and fourth saline/heroin conditioning session to index spontaneous and heroin-induced changes in ambulation, respectively. An increase in heroin-induced locomotion from injections 1–4 indicated the presence of locomotor sensitization. The time spent in the drug-paired vs. -unpaired compartment on the Post-Test served to index place-conditioning. The dose-response study of B6 mice employed 8–9/mice/group/dose, while the sample sizes employed in the single-dose study of mutant mice were: 11–15 mice/group/genotype for *Homer1a* KO, 11–3 mice/group/genotype for *Homer1* KO, 8–15 mice/group/genotype for *Homer2* KO, and 12–18 mice/group/genotype for *Grm5^R/R^* mutant.

### Surgical procedures and AAV infusion

The procedure for generating neurotropic chimeric AAV1/2 vectors carrying the renilla green fluorescent protein (hrGFP) cDNA or the hemagglutinin (HA) tag fused to the coding region of rat *Homer1c*, and *Homer2b* have been described in detail elsewhere (e.g., Klugmann et al., 2005) and the AAV-cDNA constructs were identical to those employed previously (e.g., Klugmann et al., 2005; Tappe et al., 2006; Cozzoli et al., 2009; Goulding et al., 2011; Ary et al., 2013). The design of the AAV constructs for expression of small hairpin RNAs (shRNA) against Homer1c were described in detail in Klugmann and Szumlinski (2008). Briefly, we used a bicistronic expression cassette entailing the human U6 promoter to drive the shRNA, followed by the hrGFP reporter under the control of the chicken-beta actin (CBA) promoter for identification of transduced neurons. The shRNA-Homer1c construct was the same as that used in a recently published report, in which we demonstrated approximately 50% protein knock-down within the brain at 3 weeks post-infusion (Ary et al., 2013). AAV-shEGFP-CBA-hrGFP was used as a generic control (GFP) in our AAV studies. The surgical procedures for intra-NAC AAV infusion (0.5 μl/side) were identical to those used in previous studies (e.g., Cozzoli et al., 2009) and resulted in placement of microinjectors within the boundaries of the NAC (see Figure 5A). Studies examining behavioral response in heroin-induced place-preference test after intrathecal AAV infusion employed mice whose neuropathic pain symptoms and AAV transduction patterns within spinal cord were described before (Obara et al., 2013). Following either intracranial or intrathecal infusion, animals were left undisturbed for 3 weeks when AAV-mediated transgene expression peaks to remain at maximally stable levels prior to behavioral testing (e.g., Klugmann et al., 2005; Klugmann and Szumlinski, 2008). Sample sizes employed in the statistical analyses of the data ranged from 8 to 11 mice/group/AAV for both the NAC and spinal cord study.

### Statistical analysis

Behavioral and biochemical results are presented as means ± SEM (*n* = 8–12/group). Immunoblotting data were analyzed by one-way analyses of variance (ANOVA) with Tukey’s multiple comparison *post hoc* tests and these results are presented in Table 1. Behavioral results were analyzed by two-way ANOVA and significant interactions were followed up by an analysis for simple effects and Bonferroni’s multiple comparison *post hoc* tests, when appropriate. To confirm significant place-conditioning, *a priori* dependent-sample *t*-tests were conducted for the time spent in the heroin-paired vs. -unpaired compartment, separately for each treatment group/genotype. α = 0.05 for all analyses and the results of the statistical analyses for the behavioral assays are presented in their corresponding figure legends.

**Table 1:** **Statistical results of the one-way ANOVAs conducted on the immunoblotting data (α = 0.05) and follow-up Tukey’s multiple comparison *post hoc* tests, where appropriate**.

Region	Protein	Results	
		ANOVA	*Post hoc*
PFC	mGluR1a	*F*_(2,23)_ = 10.53, *p* = 0.0007	CCI 2 weeks > CNT = CCI 1 week
	mGluR5	*F*_(2,23)_ = 3.80, *p* = 0.04	
	GluN2A	*F*_(2,21)_ = 6.47, *p* = 0.0007	CCI 1 week = CCI 2 weeks > CNT
	GluN2B	*F*_(2,23)_ = 10.39, *p* = 0.0007	CCI 2 weeks > CNT = CCI 1 week
	Homer1b/c	*F*_(2,21)_ = 13.67, *p* = 0.0002	CCI 1 week = CCI 2 weeks > CNT
	Homer2a/b	*F*_(2,19)_ = 12.51, *p* = 0.0005	CCI 1 week = CCI 2 weeks > CNT
	PKCε	*F*_(2,23)_ = 0.32, *p* = 0.73	
	pPKCε	*F*_(2,17)_ = 3.94, *p* = 0.04	CCI 2 weeks > CNT = CCI 1 week
	pPKCε:PKCε ratio	*F*_(2,17)_ = 2.06, *p* = 0.16	
	PI3K	*F*_(2,17)_ = 0.37, *p* = 0.69	
	P(Tyr)p85α	*F*_(2,19)_ = 8.22, *p* = 0.003	CCI 1 week = CCI 2 weeks > CNT
	ERK	*F*_(2,20)_ = 0.06, *p* = 0.94	
	pERK	*F*_(2,20)_ = 0.06, *p* = 0.95	
	pERK:ERK ratio	*F*_(2,17)_ = 0.01, *p* = 0.98	
NAC	mGluR1a	*F*_(2,17)_ = 1.45, *p* = 0.26	
	mGluR5	*F*_(2,17)_ = 6.97, *p* = 0.0007	CCI 1 week > CNT = CCI 2 weeks
	GluN2A	*F*_(2,17)_ = 10.52, *p* = 0.001	CCI 1 week = CCI 2 weeks > CNT
	GluN2B	*F*_(2,17)_ = 1.62, *p* = 0.23	
	Homer1b/c	*F*_(2,17)_ = 7.96, *p* = 0.004	CCI 1 week = CCI 2 weeks > CNT
	Homer2a/b	*F*_(2,17)_ = 0.24, p = 0.78	
	PKCε	*F*_(2,17)_ = 1.71, *p* = 0.21	
	pPKCε	*F*_(2,17)_ = 7.31, *p* = 0.006	CCI 1 week = CCI 2 weeks > CNT
	pPKCε:PKCε ratio	*F*_(2,17)_ = 7.28, *p* = 0.006	CCI 2 weeks > CNT = CCI 1 week
	PI3K	*F*_(2,17)_ = 0.29, *p* = 0.74	
	P(Tyr)p85α	*F*_(2,17)_ = 12.25, *p* = 0.0007	CCI 2 weeks > CNT = CCI 1 week
	ERK	*F*_(2,17)_ = 1.04, *p* = 0.38	
	pERK	*F*_(2,17)_ = 0.42, *p* = 0.67	
	pERK:ERK ratio	*F*_(2,17)_ = 7.68, *p* = 0.005	CCI 2 weeks > CNT = CCI 1 week
AMY	mGluR1a	–	
	mGluR5	*F*_(2,17)_ = 12.59, *p* = 0.0006	CCI 1 week > CNT = CCI 2 weeks
	GluN2A	*F*_(2,17)_ = 1.26, *p* = 0.31	
	GluN2B	*F*_(2,17)_ = 13.16, *p* = 0.0005	CCI 1 week = CCI 2 weeks > CNT
	Homer1b/c	*F*_(2,17)_ = 14.41, *p* = 0.0003	CCI 1 week = CCI 2 weeks > CNT
	Homer2a/b	*F*_(2,17)_ = 0.15, *p* = 0.86	
	PKCε	*F*_(2,17)_ = 12.65, *p* = 0.0006	CCI 1 week = CCI 2 weeks > CNT
	pPKCε	*F*_(2,17)_ = 16.43, *p* = 0.0002	CCI 1 week = CCI 2 weeks > CNT
	pPKCε:PKCε ratio	*F*_(2,17)_ = 0.38, *p* = 0.68	
	PI3K	*F*_(2,17)_ = 0.45, *p* = 0.64	
	P(Tyr)p85α	*F*_(2,17)_ = 13.85, *p* = 0.0004	CCI 1 week = CCI 2 weeks > CNT
	ERK	*F*_(2,17)_ = 3.83, *p* = 0.04	CCI 2 weeks > CNT = CCI 1 week
	pERK	*F*_(2,17)_ = 0.65, *p* = 0.54	
	pERK:ERK ratio	*F*_(2,17)_ = 1.69, *p* = 0.21	
VTA	mGluR1a	*F*_(2,17)_ = 3.63, *p* = 0.05	CCI 1 week = CCI 2 weeks > CNT
	mGluR5	*F*_(2,17)_ = 0.42, *p* = 0.66	
	GluN2A	*F*_(2,17)_ = 5.08, *p* = 0.02	CCI 1 week = CCI 2 weeks > CNT
	GluN2B	*F*_(2,17)_ = 6.19, *p* = 0.01	CCI 2 weeks > CNT = CCI 1 week
	Homer1b/c	*F*_(2,17)_ = 1.68, *p* = 0.22	
	Homer2a/b	*F*_(2,17)_ = 6.99, *p* = 0.007	
	PKCε	*F*_(2,17)_ = 0.17, *p* = 0.84	
	pPKCε	*F*_(2,17)_ = 6.29, *p* = 0.01	CCI 2 weeks > CNT = CCI 1 week
	pPKCε:PKCε ratio	*F*_(2,17)_ = 6.22, p = 0.01	CCI 2 weeks > CNT = CCI 1 week
	PI3K	*F*_(2,17)_ = 2.82, *p* = 0.09	
	P(Tyr)p85α	*F*_(2,17)_ = 3.87, *p* = 0.04	CCI 1 week > CNT = CCI 2 weeks
	ERK	*F*_(2,17)_ = 5.31, *p* = 0.02	CCI 1 week > CNT = CCI 2 weeks
	pERK	*F*_(2,17)_ = 0.08, *p* = 0.92	
	pERK:ERK ratio	*F*_(2,17)_ = 2, *p* = 0.005	

*The data are summarized in Figure 2 and sample sizes ranged from 6 to 8 mice/group*.

## Results

### CCI elevates mesocorticolimbic protein expression and abolishes heroin CPP

**Figure 1:**
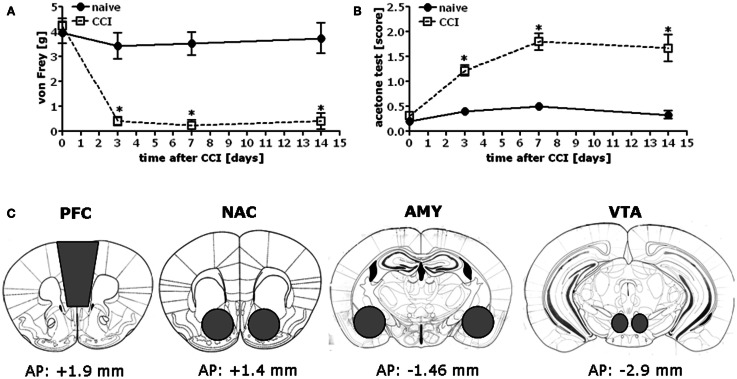
**Chronic constriction injury of the sciatic nerve results in mechanical and cold hypersensitivity in B6 mice**. When assessed at 3, 7, and 14 days post-injury, B6 mice exhibited mechanical hypersensitivity as assessed in the von Frey test **(A)** [CCI × Time: *F*_(3,56)_ = 9.07, *p* < 0.0001] and cold hypersensitivity as measured in the acetone test **(B)** [CCI × Time: *F*_(3,56)_ = 10.77, *p* < 0.0001]. The data represent the mean ± SEM of 6–8 mice/group. *p < 0.05 vs. naïve (control) mice (Bonferroni’s *post hoc* tests). **(C)** Immediately following pain threshold assessments at the 1 and 2-week time-points, tissue was obtained from the entire PFC, NAC, AMY, and the VTA of CCI and naïve mice as indicated for processing by immunoblotting.

**Figure 2:**
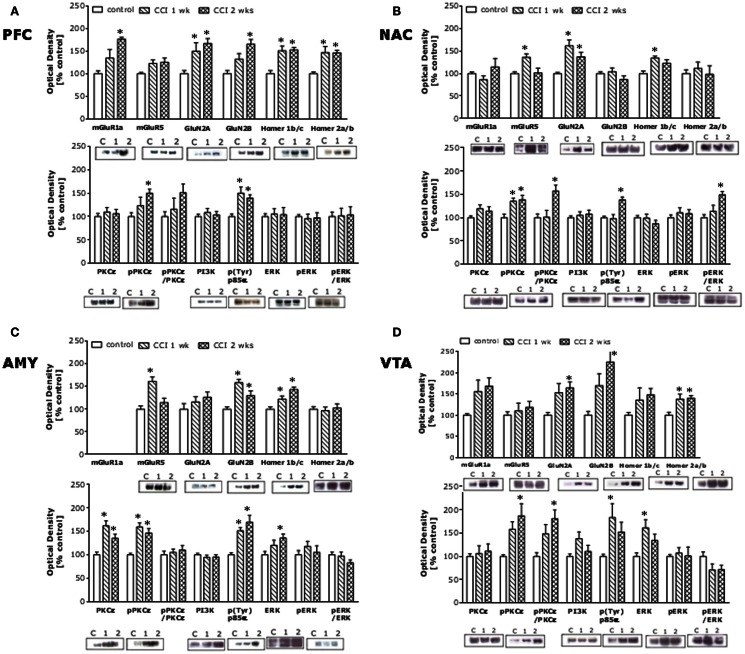
**Chronic constriction injury of the sciatic nerve augments glutamate-related protein expression throughout the mesocorticolimbic circuit**. Summary of the changes in protein expression observed within the PFC **(A)**, NAC **(B)**, AMY **(C)**, and VTA **(D)** in sciatic nerve-ligated mice (CCI) sacrificed at 1 or 2 weeks following injury, as well as in naïve controls. The following rabbit polyclonal antibodies were used: anti-Homer 2a/b and anti-Homer 1b/c (Dr. Paul F. Worley, Johns Hopkins University School of Medicine; 1:1000 dilution), anti-mGluR5 (Upstate, Lake Placid, NY, USA; 1:1000 dilution), anti-GluN2A and anti-GluN2A (Calbiochem, San Diego, CA, USA; 1:1000 dilution), anti-PI3K antibody (Upstate; 1:1000 dilution), and anti-p-(Tyr) PI3K p85α binding motif (Cell Signaling Technology, Beverly, MA, USA; 1:250 dilution), anti-ERK1/2 (Santa Cruz Biotechnology, Santa Cruz, CA, USA; 1:500 dilution), anti-PKCε and anti-p(Ser729)PKCε (Santa Cruz Biotechnology; 1:1000 dilution). Anti-mGluR1a (Upstate; 1:1000 dilution) and anti-p(Tyr204)ERK1/2 (Santa Cruz Biotechnology; 1:1000) mouse polyclonal antibodies were also used. A rabbit anti-calnexin monoclonal antibody (Stressgen, Victoria, BC, Canada; 1:1000 dilution) was used as a loading and transfer control. Immunoreactive bands were detected using enhanced chemiluminescence and immunoreactivity quantified using Image J (NIH, Bethesda, MD, USA). The data for neuropathic animals at the different time-points post-CCI were expressed as a percent change from the mean signal of the uninjured controls for each individual membrane (*n* = 3–4/membrane). The data represent the mean ± SEM of 6–8 mice/group and detailed results of the statistical analyses of these data are presented in Table 1. *p < 0.05 vs. naïve (control) mice (see Table 1; one-way ANOVA followed by Tukey’s *post hoc* tests).

Chronic constriction injury of the sciatic nerve increased mechanical and cold hypersensitivity in B6 mice (Figures 1A,B). This hypersensitivity was associated with increased expression of the majority of our proteins of interest within all four mesocorticolimbic structures investigated (as indicated in Figure 1C), with regional distinctions in the magnitude and/or time-course of the observed protein changes (Figure 2; see Table 1). In the PFC (Figure 2A), CCI increased Homer1b/c, Homer2a/b, GluN2A, and p(Tyr)p85α at both time-points post-injury, while those for mGluR1a, GluN2B, and pPKCε were time-dependent. In the NAC (Figure 2B), CCI increased Homer1b/c, GluN2A, and pPKCε at both time-points, while kinase activation increased time-dependently and the rise in mGluR5 was transient. In the AMY (Figure 2C), the rise in mGluR5 was also transient; however, CCI increased Homer1b/c, GluN2B, PKCε, pPKCε, and p(Tyr)p85α both time-points and ERK levels increased time-dependently. Unfortunately, we could not detect a reliable signal for mGluR1a within our AMY samples. Finally, in the VTA (Figure 2D), CCI increased Homer2a/b, and GluN2A at both time-points, the rise in GluN2B, pPKCε, and the pPKCε:PKCε ratio increased time-dependently and the rise in p(Tyr)p85α and ERK were transient.

**Figure 3:**
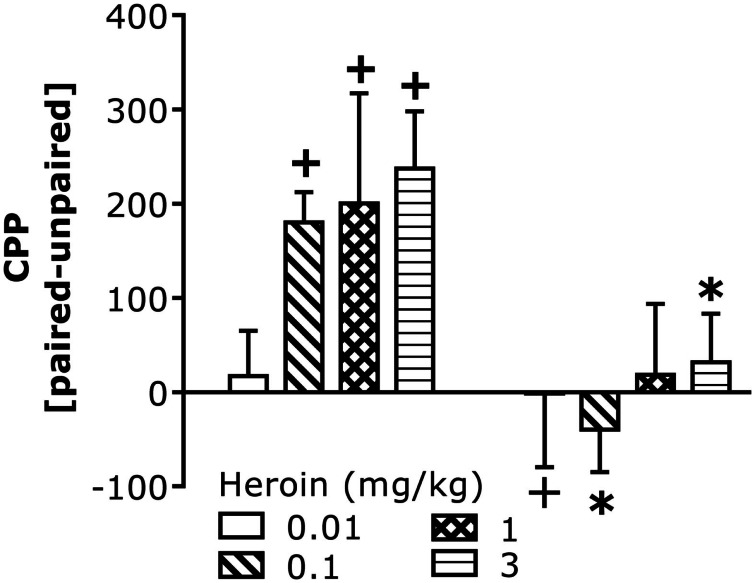
**Chronic constriction injury perturbs heroin-induced CPP in B6 mice**. Summary of the difference in the time spent (in seconds) between the heroin-paired and -unpaired compartment on a test for place-conditioning conducted 24 h following conditioning with 0.01, 0.1, 1, and 3 mg/kg heroin in B6 mice. CCI significantly altered heroin CPP [CCI × Heroin: *F*_(1,58)_ = 11.67, *p* = 0.001]. Deconstruction of the interaction along with Heroin Dose factor indicated no CCI effects at the lowest dose of heroin, but group differences at all other doses [0.01 mg/kg: *t*_(14)_ = 0.20, *p* = 0.42; 0.1 mg/kg: *t*_(16)_ = 4.63, *p* = 0.0001; 1 mg/kg: *t*_(14)_ = 1.31, *p* = 0.11; 3 mg/kg: *t*_(14)_ = 2.58, *p* = 0.01]. To confirm the presence or absence of place-conditioning in each group, *t*-tests were conducted and verified significant CPP in control naïve mice at doses of 0.1 mg/kg heroin or greater [0.1 mg/kg: *t*_(8)_ = 5.60, *p* = 0.001; 1 mg/kg: *t*_(7)_ = 2.58, *p* = 0.04; 3 mg/kg: *t*_(7)_ = 3.90, *p* = 0.006], while the 0.01-mg/kg dose produced a significant CPA in CCI mice [*t*_(9)_ = 3.97, *p* = 0.004]. No significant conditioning was observed in CCI mice at the other heroin doses (*p* > 0.05). The data represent the mean ± SEM of 8–9 animals/group/dose. *p < 0.05 vs. naïve control; +*p* < 0.05 vs. 0 min (Bonferroni’s *post hoc* tests or *t*-test).

We next assayed for CCI-induced changes in heroin-conditioned reward in B6 mice as an index of motivation. All but the lowest heroin dose elicited a significant CPP in injury-naïve B6 controls (Figure 3). In contrast, no heroin dose elicited CPP in injured B6 mice and the 0.1-mg/kg dose elicited a significant conditioned place-aversion (CPA). The injury-induced abolishment of CPP did not reflect impairments in motor activity as group differences were not observed regarding: (1) spontaneous locomotor activity (data not shown; total distance traveled during Habituation, Pre-Test, or Post-Test; *t*-tests, *p* > 0.05); (2) saline- or heroin-induced locomotor activity on injection 1 or 4; or (3) the expression of heroin-induced locomotor sensitization, which was observed only at the 3-mg/kg dose [data not shown; Heroin effect: *F*_(2,48)_ = 25.76, *p* = 0.001; Heroin × Injection: *F*_(2,48)_ = 2.87, *p* = 0.07].

### Genotype × pain interactions in heroin CPP

**Figure 4:**
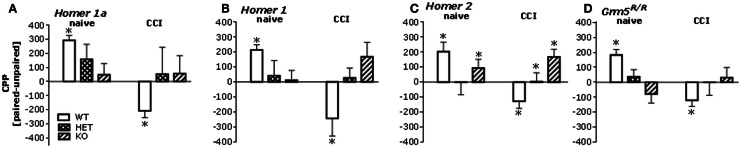
**Mutations affecting mGluR5-Homer interactions blunt heroin CPP and reverse the effects of CCI upon heroin CPA**. Summary of the difference in the time spent in the heroin-paired and -unpaired compartments (CPP) following conditioning with 0.1 mg/kg heroin in mice with constitutive deletion of *Homer1a*, *Homer1*, or *Homer2*, and in mice expressing the *Grm5^R/R^* transgene. Analysis of the data from all of the mutant animals revealed significant Genotype × CCI interactions [*Homer1a*: *F*_(2,64)_ = 3.50, *p* = 0.04; *Homer1*: *F*_(2,67)_ = 6.10, *p* = 0.004; *Homer2*: *F*_(2,66)_ = 4.14, *p* = 0.02; *Grm5^R/R^*: *F*_(2,101)_ = 6.71, *p* = 0.002]. **(A)** In uninjured mice from the *Homer1a* study, *a priori* *t*-tests (time on paired vs. unpaired side) confirmed significant CPP in *Homer1a* WT [*t*_(10)_ = 8.43, *p* < 0.0001; *n* = 11], but no place-conditioning was evidence in their HET or KO counterparts (*t*-tests, *p*’s > 0.50, *n* = 13–15). In CCI mice from the Homer1a study, CPA was apparent in WT controls (*t*_(10)_ = 2.81, *p* = 0.02; *n* = 11), but again no conditioning was apparent in their HET or KO counterparts (*t*-tests, *p* > 0.65; *n* = 8–12). **(B)** As observed in the *Homer1a* study, heroin elicited CPP and CPA, respectively in uninjured and CCI *Homer1* WT mice [naïve: *t*_(9)_ = 6.12, *p* < 0.0001; CCI: *t*_(10)_ = 2.34, *p* = 0.04], while no significant place-conditioning was apparent under either condition in HET or KO mice (*n* = 11–13; *t*-tests, *p* > 0.12). **(C)** Heroin elicited CPP and CPA, respectively, in uninjured and CCI *Homer2* WT mice [naïve: *t*_(9)_ = 3.18, *p* = 0.01; CCI: *t*_(7)_ = 2.76, *p* = 0.03]. No place-conditioning was apparent in HET mice under either condition (*n* = 15; *t*-tests, *p* > 0.90). While uninjured *Homer2* KO mice did not exhibit CPP (*n* = 11; *t*-test, *p* = 0.15), CPP, not CPA, was apparent in their CCI counterparts [*t*_(7)_ = 3.27, *p* = 0.01]. **(D)** Heroin elicited also CPP and CPA, respectively, in naïve and CCI mice *Grm5^F/^F* mice (i.e., WT) [naïve: *t*_(21)_ = 4.90, *p* < 0.0001; CCI: *t*_(18)_ = 3.00, *p* = 0.08], while no significant place-conditioning was apparent in Grm5F/R or Grm5R/R mutants (*n* = 12–18; *t*-tests, *p* > 0.20). *p < 0.05 Paired vs. unpaired (i.e., conditioning; *t*-tests); +*p* < 0.05 vs. WT control (Tukey’s *post hoc* tests).

Given the CCI-induced rise in Homer expression throughout the mesocorticolimbic system, we next assayed for low-dose heroin-induced place-conditioning in naïve and CCI *Homer1a*, *Homer1*, and *Homer2* null mutant mice, as well as in transgenic mice with a disrupted mGluR5-Homer interaction (*Grm5^R/R^*). The 0.1-mg/kg heroin dose elicited a significant CPP in injury-naïve WT mice from all strains and this CPP was absent in all homozygous mutant littermate animals (Figure 4, left). Consistent with the above data from B6 mice, the 0.1-mg/kg heroin dose elicited a significant CPA in all CCI WT mice, but this too was attenuated or prevented in all homozygous mutant mouse lines (Figure 4, right). Such data pose a necessary role for Homer1a induction, as well as scaffolding by constitutively expressed (coiled-coil) CC-Homer proteins and their interaction with mGluR5 as critical for both heroin-related appetitive and aversive learning.

### AAV-mediated homer gene transfer and injury-induced CPA

**Figure 5:**
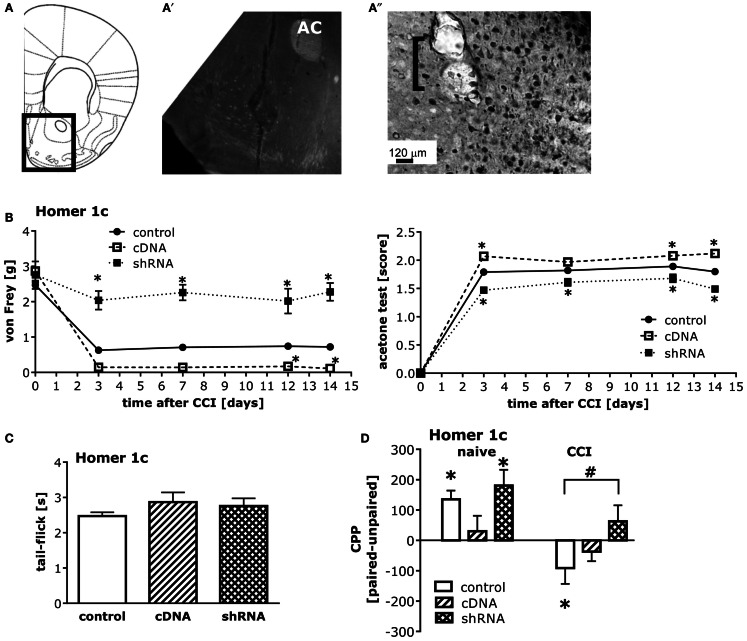
**Homer1c in the NAC bi-directionally influences neuropathic pain symptoms and heroin CPA**. **(A)** Half coronal section of the mouse brain at the level of the NAC targeted in the AAV infusion studies. **(A′)** Micrograph (4×) of GFP staining within the NAC shell produced by the shRNA-Homer1c construct [see box in **(A)** for orientation]. **(A″)** Micrograph (20×) of immunostaining for the HA-tagged cDNA-Homer1c construct within NAC shell illustrating both cell body and process staining in the tissue surrounding the microinjector tip (bracket). At 3 weeks following intra-NAC infusion, mice were subjected to CCI procedures, followed by behavioral testing. **(B)** Relative to GFP vector controls (GFP), altering NAC Homer1c expression bi-directionally altered both mechanical hypersensitivity assessed in the von Frey test [AAV × Time ANOVA: *F*_(2,130)_ = 150.6, *p* < 0.0001] and cold hypersensitivity assessed in the acetone test [AAV × Time ANOVA: *F*_(2,130)_ = 93.27, *p* < 0.0001]. *p < 0.05 vs. GFP. **(C)** No changes in the tail-flick test were observed following intra-NAC AAV infusion (one-way ANOVA, *p* = 0.38). **(D)** An AAV × CCI interaction was observed for heroin-induced place-conditioning [*F*_(2,52)_ = 3.79, *p* = 0.03]. In GFP controls, cDNA-Homer1c over-expression prevented heroin CPP, while shRNA-Homer1c was without effect [*F*_(2,28)_ = 3.36, *p* = 0.04; Tukey’s *post hoc* tests]. *T*-tests confirmed the presence of a significant CPP in GFP controls [*t*_(9)_ = 4.20, *p* = 0.002] and shRNA-infused control animals [*t*_(9)_ = 3.49, *p* = 0.007]. In contrast, both Homer1c manipulations attenuated heroin CPA in CCI mice [*F*_(2,24)_ = 7.37, *p* = 0.003; Tukey’s *post hoc* tests]. *T*-tests confirmed a significant CPA in scrambled controls [*t*_(9)_ = 3.49, *p* = 0.007], but no conditioning in Homer1c-manipulated animals (*t*-tests, *p* > 0.05). *p < 0.05 Paired vs. unpaired (conditioning; *t*-tests); ^#^*p* < 0.05 vs. scrambled AAV. The data represent the mean ± SEM of 8–11 mice/AAV/condition.

The pattern of AAV-mediated neuronal transduction within the NAC was consistent with that reported previously by our group (e.g., Cozzoli et al., 2009; Goulding et al., 2011), with little spread beyond the infusion site (Figures 5A,A′′). Intra-NAC cDNA-Homer1c and shRNA-Homer1c infusion potentiated and inhibited, respectively, both mechanical and cold hypersensitivity following CCI, but the effect was more pronounced in the von Frey test (Figure 5B). Neither Homer manipulation influenced basal pain threshold to mechanical and cold stimuli (Figure 5B) nor did they alter simple spinal pain reflex assessed in the tail-flick test (Figure 5C).

While intra-NAC shRNA-Homer1c did not influence heroin CPP in injury-naïve animals, it prevented injury-induced heroin CPA (Figure 5D, left). In contrast to shRNA-Homer1c infusion, intra-NAC cDNA-Homer1c infusion prevented heroin-induced place-conditioning in both naïve and injured groups (Figure 5D, right).

**Figure 6:**
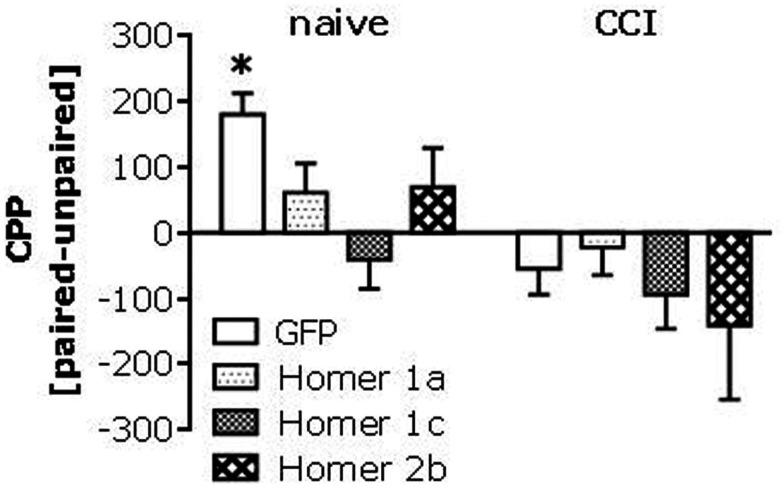
**Spinal Homer over-expression alters heroin-induced place-conditioning**. Summary of the effects of intrathecal administration of AAVs carrying cDNA for Homer1a, Homer1c, or Homer2b upon place-conditioning elicited by 0.1 mg/kg in naïve (control) and CCI mice. Analyses of these data revealed a main CCI effect [*F*_(1,70)_ = 12.82, *p* = 0.001], but interaction with the AAV factor (*p* = 0.28). *A priori* *t*-tests confirmed a significant CPP in naïve mice infused with GFP [*t*_(8)_ = 5.60, *p* = 0.001], but no CPP in any of the cDNA-infused naïve groups (*t*-tests, *p*’s > 0.05). In this study, we observed only a modest CPA in CCI-GFP controls [*t*_(8)_ = 2.05, *p* = 0.08], as well as in cDNA-Homer1c infused CCI mice [*t*_(9)_ = 1.84, *p* = 0.09], while no evidence for conditioning was observed in cDNA-Homer1a or cDNA-Homer2b CCI mice (*p*’s > 0.20). The data represent the mean ± SEM of 8–10 mice/AAV/condition. *p < 0.05 Paired vs. unpaired (conditioning; *t*-tests).

Intrathecal infusion of cDNA-Homer1c and -Homer2b potentiates, while that of cDNA-Homer1a attenuates, CCI-induced pain hypersensitivity (Obara et al., 2013). Thus, we determined whether or not spinal Homer expression might also regulate heroin place-conditioning. Intrathecal infusion of all three AAV-cDNAs blunted heroin CPP in injury-naïve mice (Figure 6, left). In this study, the heroin CPA exhibited by GFP-infused CCI mice was not as robust as that observed in the experiments above; nevertheless, none of the AAV-cDNAs influenced the extent or direction of behavior exhibited by CCI animals (Figure 6, right).

## Discussion

Pain-associated affective and motivational blunting is hypothesized to involve injury-induced changes in mesocorticolimbic function (c.f., Leknes and Tracey, 2008; Becker et al., 2012; Oluigbo et al., 2012). Thus, the present study characterized CCI-induced changes glutamate receptor expression/signaling within four major components of the mesocorticolimbic system and then assayed the functional relevance of mGluR5 interactions with its scaffolding molecule Homer (Shiraishi-Yamaguchi and Furuichi, 2007) for pain-elicited changes in heroin’s incentive motivational properties.

### Neuropathy augments indices of mesocorticolimbic glutamate transmission

Chronic constriction injury-induced hypersensitivity was associated with up-regulated mesocorticolimbic glutamate receptor and CC-Homer expression, as well as increased indices of ERK, PI3K, and/or PKCε activity. The present PFC data replicate our earlier study (Obara et al., 2013), indicating that injury up-regulates glutamate receptor signaling within a forebrain region important for volitional control over behavior, cognition, and emotion (c.f., Arnsten and Rubia, 2012; Depue, 2012). CCI-induced increases in protein expression were observed also within VTA, NAC, and AMY, with some regional differences that are not to be unexpected. However, CCI elevated Homer1b/c levels and PI3K activation in all mesocorticolimbic regions examined. Homer proteins are involved in the recruitment of PI3K-enhancer to Group1 mGluRs to induce PI3K activity (Rong et al., 2003). PI3K induction, at least within spinal cord, contributes to the development of neuropathic pain hypersensitivity (Xu et al., 2011). As an intra-NAC infusion of cDNA-Homer1c was sufficient to promote CCI-induced pain hypersensitivity, injury-induced increases in mesocorticolimbic Homer-dependent PI3K activity may contribute significantly to somatic and affective pain chronification following peripheral nerve injury. Indeed, certain AMY subregions receive direct and indirect nociceptive input from spinal cord, brainstem, thalamus, and cortex (c.f., Leknes and Tracey, 2008; Becker et al., 2012). Moreover, central sensitization, via signaling pathways involving ERK, PKCs, and PI3K, occurs within this structure in various models of chronic pain (c.f., Neugebauer et al., 2004; Neugebauer, 2006; Fu et al., 2008; Tappe-Theodor et al., 2011). Our observation of up-regulated protein expression within AMY could reflect a central sensitization of mesocorticolimbic activity that would be predicted to elicit negative emotional disturbances characteristic of chronic pain sufferers.

While we failed to detect a significant reduction in VTA ERK activity following CCI, previous studies indicated reduced VTA ERK activation and *c-fos* expression following injury, which was interpreted to reflect blunted VTA responsiveness and theorized to contribute to pain-induced amotivational states (e.g., Narita et al., 2003, 2004; Ozaki et al., 2004). However, CCI elevated our other indices of signaling within VTA, most notably GluN2 subunits, Homer2a/b, activated PKCε, and PI3K, which would be predicted to elevate, rather than depress, *basal* activity of mesolimbic dopamine neurons to heighten the saliency of both conditioned and unconditioned pain cues (Berridge, 2007; Bromberg-Martin et al., 2010). Indeed, these present immunoblotting results are consistent with human neuroimaging data indicating correlations between heightened PFC-NAC connectivity and pain chronification (Baliki et al., 2010, 2012). Thus, injury-induced plasticity within corticofugal glutamatergic and mesocorticolimbic dopaminergic projections might heighten PFC-NAC connectivity predictive of somatic and affective pain chronification. In support of this notion, NAC Homer1c expression bi-directionally altered CCI-induced pain symptoms, with increased Homer1c promoting nociception in CCI mice (see below).

### Heroin CPP and Homer-mGluR5 interactions

In all experiments, repeated low-dose (0.1 mg/kg) heroin consistently supported CPP in injury-naïve WT mice. Remarkably, this low-dose heroin CPP was attenuated or absent in injury-naïve mice from all four mutant strains. Opioids and their withdrawal alter *Homer1* gene products within the PFC and AMY (Ammon et al., 2003; Kuntz et al., 2008) and recently, polymorphisms in *Homer1*, as well as changes in striatal and AMY *Homer1* mRNA expression, were reported in post-mortem studies of heroin addicts (Okvist et al., 2011; Jacobs et al., 2012). While constitutive *Homer2* deletion does not impact heroin-induced locomotor activity (Szumlinski et al., 2004), to the best of our knowledge, these data are the first to describe the heroin reward phenotype produced by constitutive deletion of different *Homer* genes or transgenic disruption of mGluR5-Homer interactions. That null mutations of *Homer1a* and *Homer1* (the latter of which eliminates both inducible and CC Homer1 isoforms; see Yuan et al., 2003) produced a more pronounced effect upon conditioning than *Homer2* deletion argues a more critical role for *Homer1* gene products, particularly Homer1a, in this form of heroin-related learning. Moreover, the fact that *Grm5^R/R^* mice not only failed to exhibit heroin CPP, but tended toward CPA, argues further that the interaction between *Homer1* gene products and mGluR5 is fundamental to the motivational valence of low-dose heroin, which is worthy of further exploration. The *Grm5^R/R^* data are interesting as the effect of mGluR5 antagonism upon opioid-induced CPP is inconsistent (Popik and Wróbel, 2002; McGeehan and Olive, 2003; van der Kam et al., 2009). As the *Grm5^R/R^* mutation does not impact total receptor expression (Cozzoli et al., 2009), the present behavioral observations implicate intracellular signaling processes that are known to be modulated by dynamic changes in Homer1a/CC-Homer interactions with mGluR5 in the positive incentive motivational properties of heroin-paired cue/contexts. Such signaling processes include (but are not likely limited to): altered regulation of voltage-gated ion channels, constitutive mGluR5 activity, induction of PI3K activity, and mGluR-dependent regulation of NMDA receptor current (c.f., Shiraishi-Yamaguchi and Furuichi, 2007). While the precise biochemical mechanisms mediating the blunted heroin CPP exhibited by *Homer* mutant and *Grm5^R/R^* mice obviously require detailed study that are beyond the scope of this report, the results of larger-scale dose-response studies of cocaine or alcohol CPP argue that this heroin phenotype does not reflect a mere impairment of associative learning processes (Szumlinski et al., 2004, 2005; Datko et al., 2008; Goulding et al., 2009). Unfortunately, cessation of breeding programs for the various mutant lines precludes a full dose-response analysis of heroin CPP. Thus, it remains to be determined whether or not the blunted low-dose heroin CPP observed in injury-naïve *Homer1a/1/2* or *Grm5^R/R^* mutant mice reflects changes in the sensitivity or efficacy of heroin to elicit conditioned reward or if the blunted CPP extends to any other measure of heroin reward/reinforcement. However, arguing against increased sensitivity to heroin intoxication as a mechanism underpinning the blunted heroin CPP, all mutant lines exhibited WT-levels of heroin-induced locomotion throughout testing.

Interestingly, *Homer1* deletion abolished low-dose heroin CPP, while intra-NAC shRNA-Homer1c infusion had absolutely no effect. These data indicate either that: (1) the neural locus mediating the CPP effect of *Homer1* deletion resides outside the NAC or (2) the CPP effect of *Homer1* deletion reflect an absence of inducible, rather than constitutively expressed, *Homer1* gene products. As the effects of *Homer1a* deletion mirrored those of *Homer1* deletion argues in favor of the latter possibility. However, based on suggestions of regional differences in heroin-induced changes in *Homer1* mRNA within PFC, AMY, and dorsal striatum (Kuntz et al., 2008; Okvist et al., 2011; Jacobs et al., 2012), *Homer1* gene products in these other addiction-relevant brain regions may contribute more so to the conditioned incentive motivational properties of low-dose heroin. It is interesting to note, however, that intra-NAC cDNA-Homer1c, as well as intrathecal cDNA-Homer1a, -Homer1c, and -Homer2b infusion, in injury-naïve mice was sufficient to block heroin CPP. The result for the NAC may be counterintuitive based on the findings from the KO studies, but, as argued below, may reflect a facilitation of low-dose heroin hyperalgesia that renders the heroin experience more aversive.

### Injury-induced heroin CPA also requires intact mGluR5-Homer interactions

Most notable and distinct from the results of earlier CPP studies in injured animals (c.f., Niikura et al., 2010), neuropathic B6 mice exhibited CPA in response to 0.1 mg/kg heroin – a dose of heroin that supported CPP in uninjured animals. In WT mice, CCI clearly augmented pain symptoms prior to heroin conditioning (see also Obara et al., 2013), supporting a causal relation between pain symptomatology and low-dose heroin aversion. In further support of a direct cause-effect relation between pain and heroin aversion, cDNA-Homer1 infusion into either the NAC or spinal cord augments pain hypersensitivity and abolishes heroin CPP in injury-naive animals. Furthermore, intra-NAC shRNA-Homer1c infusion, a manipulation that reduced pain hypersensitivity following CCI, prevented subsequent heroin CPA. However, neither intra-NAC nor intrathecal cDNA-Homer1c infusion potentiated heroin CPA in CCI animals. In fact, NAC cDNA-Homer1c transduction in CCI mice attenuated heroin CPA, although the magnitude of place-conditioning was not statistically different from GFP-infused CCI controls. These data, coupled with the lack of any significant cDNA effect in our spinal cord study (where weak CPA was observed in CCI mice) argue against a ceiling effect limiting the expression of CCI-induced CPA. Arguably, however, the fact that the effects of cDNA-Homer infusion upon heroin place-conditioning were not additive with those produced by CCI alone might be interpreted to reflect mechanistic interdependency, an interpretation that would be consistent with the notion that CCI-induced increases in glutamate receptor/Homer expression are neuroadaptations that promote dysphoric states.

Indeed, the present data from the studies of transgenic mice support this possibility as no evidence for CCI-induced heroin CPA was apparent in any mutant strain; in fact, both *Homer1* and *Homer2* KO mice exhibited conditioned approach behavior following nerve injury. That both *Homer1a* deletion and the *Grm5^R/R^* transgene exacerbate neuropathic pain symptoms, while neither *Homer1* nor *Homer2* deletion influence pain hypersensitivity (Obara et al., 2013), argues that the severity of neuropathic pain symptoms is not a determinant of CCI-induced deficits in heroin CPP (Table 2). CCI-induced neuropathy likely involves temporally dynamic changes in inducible vs. constitutive Homer expression, with early post-injury elevations in inducible Homers facilitating synaptic rearrangement that is later maintained by injury-induced increases in CC-Homer expression (e.g., Miletic et al., 2005, 2009; Miyabe et al., 2006; Tappe et al., 2006; Ma et al., 2009; Obara et al., 2013). Thus, the genetic interruption of the temporal dynamics of the interplay between inducible and CC-Homer protein expression at glutamate receptors, and likely other Homer-interacting molecules, while not always sufficient to prevent neuroplasticity within pain pathways, appears to be sufficient to prevent whatever mesocorticolimbic neuroplasticity mediating CCI-induced deficits in heroin-conditioned reward. Given the present data, it becomes important to characterize more systematically: (1) how heroin dose interacts with a chronic pain state to influence drug reward/reinforcement and to relate these interactions to the expression of different Homer isoforms, as well as their major interacting partners throughout the central nervous system; (2) to extrapolate findings for heroin to prescription opioid drugs employed in pain management, and importantly; (3) to examine the relevance of injury-induced changes in glutamate receptor/Homer expression for the incentive motivational properties of opioid and other non-opioid analgesic drugs (e.g., cannabinoids). Arguably, such lines of investigation will enable a better understanding of the molecular and cellular processes mediating pain-induced dysphoria, which has relevance not only for therapeutic intervention of pain-induced negative affective states, but also individual vulnerability to develop abuse or addiction during pain management with opioid or non-opioid drugs with high abuse potential.

**Table 2:** **Comparison of the effects of constitutive gene mutations affecting mGluR5-Homer interactions or AAV-mediated changes in Homer expression upon the development of neuropathic pain symptoms following CCI, the expression of a low-dose heroin CPP, and the heroin CPA observed in CCI animals (present study; Obara et al., 2013)^1^**.

Gene manipulation	CCI pain symptoms	Heroin CPP	CCI-induced heroin CPA
**CONSTITUTIVE GENE MUTATION**			
*Homer1a* KO	↑^1^	↓	↓
*Homer1* KO	No effect^1^	↓	↓
*Homer2* KO	No effect^1^	↓	↓ (Full reversal)
*Grm5^R/R^*	↑^1^	↓	↓
**AAV-MEDIATED GENE TRANSFER**			
NAC cDNA-Homer1c	↑	↓	↓
NAC shRNA-Homer1c	↓	No effect	↓
IT cDNA-Homer1a	↓^1^	↓	No effect
IT cDNA-Homer1c	↑^1^	↓	No effect
IT cDNA-Homer2b	↑^1^	↓	No effect

*CCI, chronic constriction injury of the sciatic nerve; CPA, conditioned place-aversion; CPP, conditioned place-preference; IT, intrathecal; NAC, intra-nucleus accumbens*.

## Conflict of Interest Statement

The authors declare that the research was conducted in the absence of any commercial or financial relationships that could be construed as a potential conflict of interest.
